# Alternative splicing: an underexplored layer in immune receptor regulation, systemic resistance and priming

**DOI:** 10.3389/fpls.2026.1756671

**Published:** 2026-03-13

**Authors:** Federico E. Aballay, Iván J. León Sánchez, Camila Benelli, Suruchi Roychoudhry, Damián A. Cambiagno, Ezequiel Petrillo, Nicolás M. Cecchini

**Affiliations:** 1 Instituto de Fisiología, Biología Molecular y Neurociencias (IFIBYNE), CONICET-Universidad de Buenos Aires, Buenos Aires, Argentina; 2 Universidad Nacional de Buenos Aires, Facultad de Ciencias Exactas y Naturales, Departamento de Fisiología, Biología Molecular y Celular, Buenos Aires, Argentina; 3 Universidad Nacional de Córdoba, Facultad de Ciencias Químicas, Departamento de Química Biológica Ranwel Caputto, Córdoba, Argentina; 4 CONICET, Universidad Nacional de Córdoba, Centro de Investigaciones en Química Biológica de Córdoba (CIQUIBIC), Córdoba, Argentina; 5 Centre for Plant Sciences, University of Leeds, Leeds, United Kingdom; 6 Unidad de Estudios Agropecuarios (UDEA), INTA-CONICET, Córdoba, Argentina

**Keywords:** alternative splicing, Arabidopsis, chromatin–splicing crosstalk, exitron splicing, intron retention, PRR and NLR receptors, plant immunity, priming

## Abstract

Plant immunity relies on precise regulation of pattern-recognition receptors (PRRs) and nucleotide-binding leucine-rich repeat receptors (NLRs). Beyond triggering local defenses, these receptors also induce durable systemic resistance, establishing an immune memory or “primed” state that enables faster and stronger responses upon re-infection. While chromatin-based mechanisms are well-recognized contributors to systemic resistance and priming, emerging evidence suggests that alternative splicing (AS) may provide an additional, largely overlooked regulatory layer. AS is reprogrammed during pathogen attack and reshapes both the quantitative and qualitative expression of many defense components, including *PRRs*, *NLRs*, downstream kinases, and splicing regulators. In this Perspective, we present a hypothesis-generating computational study based on the integrative reanalysis of publicly available transcriptomic datasets. By combining priming and splicing-associated transcriptomes with an improved isoform-resolved reference transcriptome, we explore AS regulation within *PRR* and *NLR* gene families. This approach highlights priming-associated AS patterns affecting subsets of immune receptors, supporting the hypothesis that AS-mediated modulation of receptor isoform pools may contribute to immune priming. We further propose that AS could generate functionally distinct receptor isoforms, providing a mechanism for immune receptors’ plasticity without constitutive defense activation. Finally, we outline experimental strategies required to validate these computational predictions and to define isoform-specific functions of immune receptors in plant immune memory.

## 1 Introduction

As sessile organisms, plants cannot escape stress nor pathogens and must reprogram their transcriptome to balance growth and defense. Their innate immune system relies on the recognition of foreign molecules (Spoel and Dong, 2012; Jones et al., 2024). Two main receptor classes mediate pathogen detection: pattern recognition receptors (PRRs) at the plasma membrane, which sense pathogen-associated molecular patterns (PAMPs) such as bacterial flagellin (Macho and Zipfel, 2014), and intracellular nucleotide-binding leucine-rich repeat receptors (NLRs) that recognize, directly or indirectly via host target modifications, pathogen effectors promoting virulence (Jones et al., 2016). These receptors activate pattern-triggered immunity (PTI) or effector-triggered immunity (ETI), respectively (Jones and Dangl, 2006). PTI and ETI converge on shared downstream outputs, including mitogen-activated protein kinase (MAPK) activation, Ca²^+^ influx, reactive oxygen species (ROS) production, and extensive transcriptional reprogramming, enhancing each other to confer full immunity (Ngou et al., 2021; Bernoux et al., 2022; Chen et al., 2022; Yu et al., 2024).

PRR immune complexes comprise receptor-like kinases (RLKs) and receptor-like proteins (RLPs) located in the plasma membrane; RLKs contain an intracellular kinase domain, whereas RLPs depend on co-receptors such as SERK family members (e.g., BAK1) or SOBIR1 to initiate signaling (Couto and Zipfel, 2016). Receptor-like cytoplasmic kinases (RLCKs) also act closely downstream of PRRs, directly linking receptor activation to early defense responses (DeFalco and Zipfel, 2021). On the other hand, NLRs constitute one of the largest gene families in plants, and typically comprises an N-terminal domain, a nucleotide-binding (NB-ARC) domain, and a leucine-rich repeat (LRR) region (Cesari, 2018; Contreras et al., 2023). Flowering plants contain three major NLR groups based on their N-terminal domains: “Toll/Interleukin-1 receptor” NLRs (TNLs), “coiled-coil” NLRs (CNLs), and “RPW8-like coiled-coil” NLRs (RNLs), named after the *Resistance to Powdery Mildew 8* (RPW8) domain (Kourelis et al., 2021; Chia and Carella, 2023). In addition, based on their functionality, NLRs can be classified into “sensor” NLRs that detect pathogen effectors, and “helper” NLRs, such as the Arabidopsis RNLs of the ADR1 and NRG1 families, which act downstream of multiple sensor NLRs to transduce and amplify immune signaling (Jubic et al., 2019; Gong et al., 2023). Activated NLRs can assemble into oligomers, the so-called resistosomes, with some of them forming plasma membrane pores that mediate Ca²^+^ influx, defenses signaling, and cell death (Huang et al., 2023; Shepherd et al., 2023).

Pathogen perception by both PRR and NLRs not only triggers local defenses but also induces durable systemic resistance, usually establishing an immune memory or “primed” state that enables faster and stronger responses to subsequent infections or secondary challenges (Martinez-Medina et al., 2016; Conrath, 2025). This physiological memory increases plant fitness under biotic stress. Systemic resistance can arise from local stimuli in leaves or roots, activating different induced systemic resistance (ISR) programs (De Kesel et al., 2021). Among these, systemic acquired resistance (SAR) is one of the best-characterized forms associated with priming and is triggered by leaf infection with necrotizing pathogens (Fu and Dong, 2013; Conrath et al., 2015). In addition, priming can also be established by defense-related mobile metabolites such as azelaic acid (AZA), and pipecolic acid (PIP) or its active derivative N-hydroxy-PIP (NHP) (Zimmerli et al., 2000; Jung et al., 2009; Navarova et al., 2012; Chen et al., 2018; Hartmann and Zeier, 2018; Vlot et al., 2021; Honig et al., 2023).

Primed plants retain molecular information without constitutively expressing defenses (Martinez-Medina et al., 2016). This memory involves chromatin remodeling, accumulation of inactive signaling components (e.g., MAPKs), and changes in receptor abundance or localization (Beckers et al., 2009; Tateda et al., 2014; Cecchini et al., 2015; Tsuda and Somssich, 2015; Baum et al., 2019). Particularly, epigenetic mechanisms (e.g. DNA methylation, histone modification, and RNA-directed DNA methylation (RdDM)) are central in maintaining primed states (Jaskiewicz et al., 2011; Luna and Ton, 2012; Singh et al., 2014; Wilkinson et al., 2019; Conrath, 2025). In Arabidopsis, chromatin remodelers such as DECREASE IN DNA METHYLATION 1 (DDM1) and “MORPHEUS’ MOLECULE 1 (MOM1) play important roles in maintaining and/or resetting primed states through regulation of PRR and NLR (Alvarez et al., 2010; Cambiagno et al., 2018; Furci et al., 2019; Cambiagno et al., 2021). Both factors act as negative regulators of priming, maintaining receptors clusters silenced and preventing transgenerational memory (Iwasaki and Paszkowski, 2014; Cambiagno et al., 2021; Miranda de la Torre et al., 2023). While DDM1 has broader effects along chromosomes, MOM1 is recognized to be less pleiotropic, affecting only a subset of pericentromeric loci and without disrupting DNA methylation and other epigenetic features (Habu et al., 2006; Vaillant et al., 2006; Zemach et al., 2013; Lyons and Zilberman, 2017; Akinmusola et al., 2023; Li et al., 2023). Even though epigenetic and chromatin-remodeling components underlying primed states are partly understood (Crisp et al., 2016; Mozgova et al., 2018; Alonso et al., 2019; Cambiagno et al., 2021; Hannan Parker et al., 2022; Manuel et al., 2025) other potential factors/mechanisms of immune memory remain largely overlooked.

Beyond chromatin-based mechanisms, RNA-level regulation may constitute an additional layer of immune priming. Among these processes, alternative splicing (AS) remains underexplored, despite its ability to generate multiple transcript isoforms that could diversify the regulatory activity of immune components (Kornblihtt et al., 2013; Allemand et al., 2016; Agirre et al., 2021). Evidence from thermopriming, for example, shows extensive AS reprogramming upon re-challenge (Ling et al., 2018). Thus, AS could help maintain the immune-primed state by producing receptor isoforms with distinct activities and/or by modulating splicing regulators such as serine-arginine-rich (SR) proteins and heterogeneous nuclear ribonucleoproteins (hnRNPs), thereby reconfiguring the transcriptome during secondary challenges (Yang et al., 2014; Borrelli et al., 2018; Hewezi, 2024; Godinho et al., 2025). Furthermore, the co-transcriptional nature of splicing sets the scene for coupling, i.e. a bidirectional relationship between chromatin and splicing. Whilst histone modifications and chromatin density dictate alternative splicing decisions co-transcriptionally, the recruitment of the splicing machinery conversely acts as a feedback mechanism that recruits ‘writers’ to modify and maintain the local epigenetic landscape (Kornblihtt et al., 2013; Allemand et al., 2016; Whittaker and Dean, 2017; Agirre et al., 2021). Thus, the AS process could also modulate chromatin-linked priming by acting as a functional scaffold that recruits epigenetic modifiers, thereby establishing a persistent chromatin state that facilitates rapid transcriptional reprogramming upon subsequent pathogen challenges. By adjusting isoform composition, AS may enable plants to remain in a poised yet energy-efficient defensive state. This may be particularly relevant for immune receptors, whose modular architecture and diversity suggest potential for transcript-based regulation (Yang et al., 2014; Borrelli et al., 2018; Hewezi, 2024; Godinho et al., 2025). Moreover, given the central role of PRRs and NLRs in defense, AS emerges as a compelling mechanism for modulating their function.

In this Perspective, we build on this hypothesis through a computational reanalysis of publicly available transcriptomes, integrating priming- and splicing-associated datasets with the Reference Transcript Dataset for Arabidopsis 2 (AtRTD2_QUASI), which enables improved resolution of alternative transcript isoforms (Zhang et al., 2017a). We use these data to evaluate whether AS may shape PRR and NLR isoform profiles across primed states, secondary challenges, and perturbations of splicing. This *in silico* analysis supports the idea that AS represents an overlooked but potentially important regulatory layer in plant immunity and systemic resistance–associated immune priming. Importantly, it also allows us to outline experimental strategies required to validate these hypotheses and to guide future studies of isoform-specific functions in plant immune memory.

## 2 Alternative splicing-driven immune receptor reprogramming during priming

### 2.1 Alternative splicing as a regulatory layer in plant defenses

Alternative pre-mRNA splicing (AS) is an RNA processing event and is a major co-/post-transcriptional mechanism for shaping plant stress responses (Staiger and Brown, 2013). By generating multiple transcript isoforms through alternative 5’ or 3’ splice sites, exon skipping, intron retention (IR) and exitron splicing (EIS), AS modulates mRNA abundance, protein diversity, and regulatory flexibility (Kornblihtt et al., 2013; Chaudhary et al., 2019; Marquardt et al., 2023). IR is the predominant AS class in plants, frequently producing transcripts with premature termination codons (PTCs) subjected to nonsense-mediated mRNA decay (NMD) or alternative polyadenylation, thereby finely tuning transcript stability and translation (Petrillo, 2023; Tognacca et al., 2023). Previously buried in the category of (cryptic) intron retention events, exitrons represent a functionally distinct AS event type that can remodel protein domain composition while preserving coding potential (Marquez et al., 2015; Ghelli et al., 2018; Cheng et al., 2020; Cecchini et al., 2022).

During biotic stress, AS is extensively reprogrammed and affects receptors, kinases, transcription factors, and RNA surveillance components, underscoring its broad contribution to immune regulation (Yang et al., 2014; Filichkin et al., 2015; Kufel et al., 2022). For example, *Pseudomonas syringae* infection has been proposed to widely alter AS of Arabidopsis genes (Howard et al., 2013). Several splicing factors, including SR proteins, hnRNPs, Sm/LSm proteins, ZINC-FINGER AND OCRE DOMAIN PROTEIN 1 (ZOP1), ARGININE/SERINE-RICH SPLICING FACTOR 45 (SR45), MODIFIER OF SNC1-1 (MOS14), SMALL NUCLEAR RIBONUCLEOPROTEIN 13 (SNU13), PROTEIN ARGININE METHYLTRANSFERASE 5 (PRMT5), and ION CHLORIDE NUCLEOTIDE-SENSITIVE PROTEIN (PICLN), modulate AS of defense-associated transcripts and influence local and/or systemic resistance (Sanchez et al., 2010; Xu et al., 2011; Zhang et al., 2017b; Bazin et al., 2020; Mateos et al., 2023; Agrofoglio et al., 2024). Additional spliceosome-associated components, including NTC-related factors (NTR1, ILP1) and RS/SR proteins (RS31, SR34a) also regulate AS of defense genes and/or stress-related pathways (Palusa et al., 2007; Duque, 2011; Laloum et al., 2018; Wang et al., 2019; Koster et al., 2025). Thus, AS, across its different modes, regulates primary local defense responses, and the splicing regulators appear to play central roles. This provides a plausible mechanism through which AS could also modulate systemic resistance programs and associated priming.

### 2.2 AS in Arabidopsis PRR and NLR immune receptors

Balanced *PRR/NLR* expression is critical, particularly for NLRs, whose overaccumulation can trigger autoimmunity, whereas insufficient diversity restricts pathogen recognition. Their abundance, localization, and functional plasticity are regulated through transcriptional control, alternative transcription start sites (TSSs), and, importantly, alternative splicing (Lai and Eulgem, 2018; Rigo et al., 2019; Kourelis and Adachi, 2022; Shepherd et al., 2023; Sun et al., 2024; Peppino Margutti et al., 2025). Although only a subset of Arabidopsis *PRRs* and *NLRs* (e.g. *RPS4*, *SNC1*, *N*, *FLS2*, *RAC1*) have experimentally validated AS events, several reviews emphasize the diversity of AS across immune receptors (Staiger and Brown, 2013; Yang et al., 2014; Lai and Eulgem, 2018; Kufel et al., 2022; Zhu et al., 2025). Nevertheless, the full scope and functional relevance of AS for defense/receptors signaling remains largely unexplored (Zhang et al., 2014; Staiger and Simpson, 2015; Chaudhary et al., 2019; Liu et al., 2024; Alhabsi et al., 2025).

**Figure 1:**
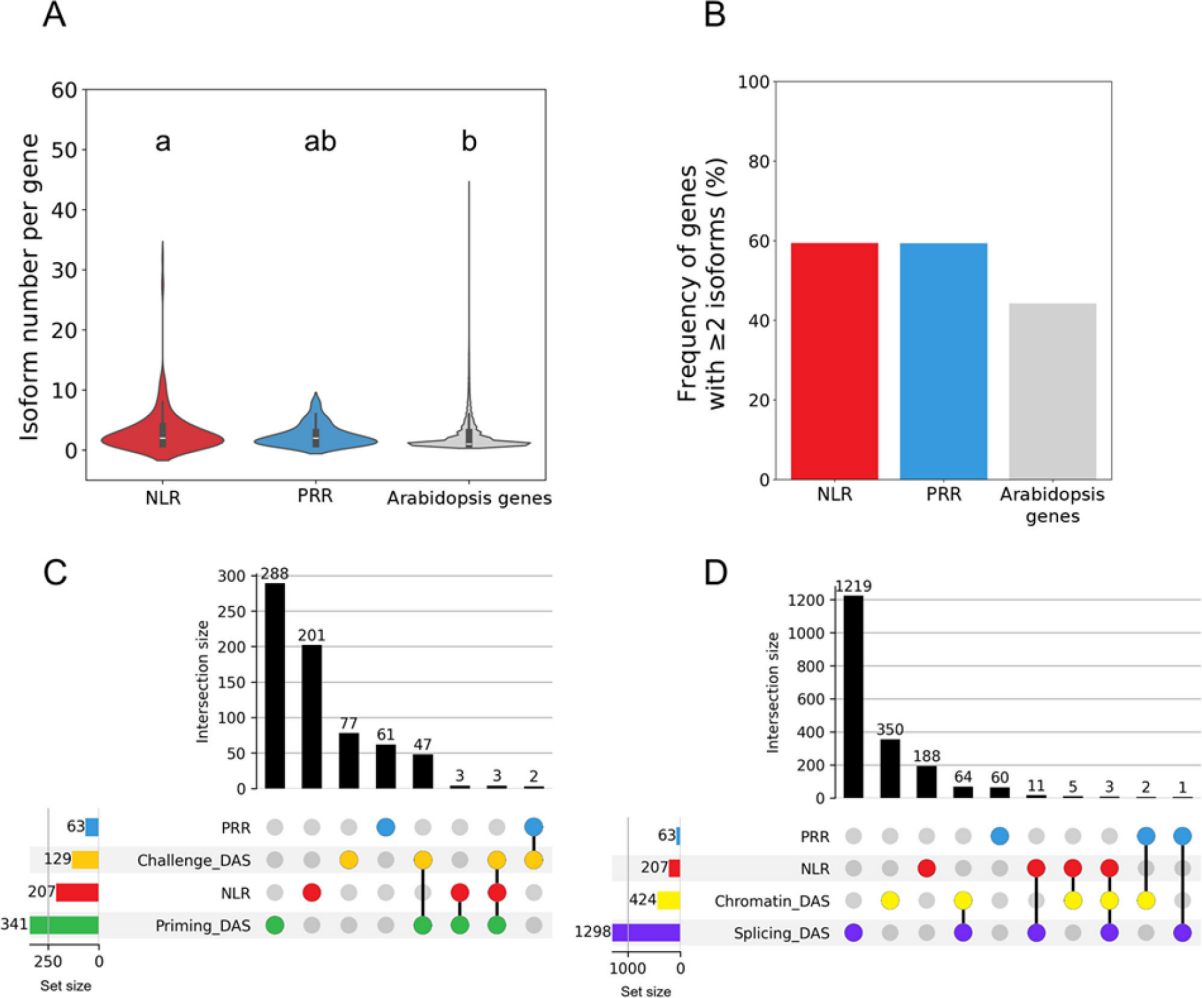
Isoform potential and priming-driven AS remodeling in Arabidopsis immune receptors. **(A)** Number of annotated alternative isoforms of immune receptors in AtRTD2. Note that NLRs produce more isoforms than PRRs and all Arabidopsis genes. Different letters indicate significant differences using the Kruskal-Wallis test (p<0.01). **(B)** Frequency of genes with two or more isoforms from the subsets of NLRs and PRRs in comparison to all Arabidopsis genes. Both receptor groups likely undergo AS to a higher extent than the Arabidopsis transcriptome (Total genes). **(C)** UpSet plot illustrating the intersection between differentially alternatively spliced (DAS) events detected during primed-state maintenance (Priming_DAS) or after secondary challenge (Challenge_DAS) and curated sets of immune receptors (PRRs and NLRs) in chitin-primed plants (Makechemu et al., 2025). **(D)** UpSet plot illustrating the overlap between DAS events triggered by mutations in splicing regulators (*prmt5, picln, ilp1, RS31OX*; Splicing_DAS) or chromatin remodelers (*mom1, ddm1*; Chromatin_DAS) and curated PRR/NLR gene lists (Wang et al., 2019; Ning et al., 2020; Mateos et al., 2023; Koster et al., 2025). In both UpSet plots, rows represent gene sets and colored dots indicate their participation in each intersection; connected dots define the specific set combination. Vertical bars show the number of genes in each intersection, while horizontal bars indicate total set sizes. Colors are used to distinguish gene categories or regulatory conditions and are consistent across panels.

This gap in knowledge about the impact of AS in immune receptors, particularly regarding how frequently and to what extent *PRRs* and *NLRs* undergo AS, motivated us to systematically analyze AS signatures across these receptor families. To indirectly assess their potential for AS-mediated regulation, we examined total isoform numbers for Arabidopsis *NLR* and *PRR* genes (**Supplementary Figure 1**). PRR components, including RLKs, RLPs, SERKs, and PTI-associated RLCKs, were compiled from validated amiGO database and PTI networks. Then, a NLR set was assembled using NLRscape database (Martin et al., 2023). Finally, both groups were analyzed for isoform diversity in the same manner (**Supplementary Tables 1**-**4**). Notably, as shown in **Figure 1A**, NLRs exhibit a substantially higher number of isoforms per gene. Importantly, this pattern is not driven by a few outlier genes producing unusually large isoform repertoires; rather, it reflects a consistent trend across both receptor families (i.e. NLRs and PRRs) towards widespread alternative processing (**Figure 1B**). This pattern supports the idea that NLRs, as highly variable intracellular receptors dedicated to effector recognition, may maintain a greater potential for diversification via alternative isoforms. In contrast, the lower isoform diversity among PRRs is consistent with their function in sensing conserved microbial patterns, which may reduce the need for extensive isoform modulation. Together, these observations highlight the regulatory potential of AS in immune receptors, particularly in the *NLR* loci, and underscore the need to further investigate how isoform dynamics shape receptor responsiveness, contribute to immune plasticity, and participate in immune priming.

### 2.3 Assessment of gene expression and alternative splicing of immune receptors

Building on the extensive isoform diversity observed in immune receptors (**Figures 1A, B**), we asked whether immune priming induces specific AS adjustments, whether splicing regulators contribute, or whether chromatin remodelers, previously implicated in priming, influence PRR/NLR AS. To address these questions, we implemented a computational pipeline using AtRTD2_QUASI (**Supplementary Figure 1**). Raw sequencing reads from publicly available datasets were downloaded, adapter-trimmed, and quantified using Salmon (Patro et al., 2017) against this reference. Transcript-level quantifications were analyzed using 3D RNA-seq App (Calixto et al., 2018; Guo et al., 2021). Read counts and transcripts per million (TPM) values were imported and summarized with the R package tximport v1.10.0 using the *lengthScaledTPM* method (Soneson et al., 2016). Lowly expressed transcripts were filtered out based on the mean-variance trend of the data; transcripts were considered expressed if they showed counts per million (CPM) ≥ 2 in at least two samples, and genes were defined as expressed when at least one transcript met this criterion. Gene and transcript counts were normalized using the TMM method (Bullard et al., 2010). Differential gene expression (DGE) and differential alternative splicing (DAS) analyses were performed using thresholds of |log_2_ (fold change)| ≥ 1 and |ΔPS| ≥ 0.15, respectively. P-values were corrected for multiple testing using the Benjamini–Hochberg procedure, and genes were considered significantly differentially expressed or spliced at a false discovery rate (FDR) < 0.05.

### 2.4 AS during primed state maintenance and secondary challenge

To evaluate AS-mediated changes in *PRR* and *NLR* immune receptors during the primed state, either during memory maintenance or during the enhanced response to a secondary challenge, we compared transcriptomic data from primed and non-primed Arabidopsis plants. Among publicly available studies, we selected the chitin-induced systemic priming dataset from Makechemu et al. (2025), as it retains the largest number of expressed *PRR*/*NLR* receptor transcripts suitable for analysis (86% of total receptors), minimizing underestimation of receptor isoform diversity (**Figure 1C**, **Supplementary Table 5**).

We used data from Arabidopsis aerial tissues of plants whose roots were watered with chitin (primed state) or only water (naïve), and from plants subsequently challenged in leaves with *Pseudomonas syringae* pv. *tomato* DC3000 (*Pst*) or mock-inoculated (secondary challenge) (Makechemu et al., 2025). We then quantified DAS to identify receptor isoforms enriched in primed tissues or specifically induced upon secondary challenge. Comparisons between primed and naïve plants (Priming_DAS) and after secondary challenge (Challenge_DAS) revealed distinct AS signatures, indicating that some PRRs, and especially NLRs, undergo AS modulation (**Figure 1C**). Only two PRRs showed DAS specifically upon challenge, *EFR* and *LORE/SD1-29*-, both core PTI receptors (Macho and Zipfel, 2014; Ranf et al., 2015) (**Table 1**). Among the six NLRs with DAS, several corresponded to well-characterized receptors (e.g. *RPP7*, *CHS2*/*RPP4*, *RPP13*; (Bittner-Eddy et al., 2000; van der Biezen et al., 2002; Li et al., 2020). Three showed DAS only during primed state maintenance, and the remaining three were detected in both memory maintenance and secondary challenge phases.

**Table 1:** Differentially alternatively spliced (DAS) genes identified across Arabidopsis transcriptomes from primed tissues and from plants with perturbed splicing factors or chromatin remodelers.

Gene ID	Gene name	Receptor class^a^	Subclass^b^	Source dataset^c^	Condition^d^	AS events detected^e^	Has any NMD-targeted isoform?^f^
*AT1G58602*	*RPP7*	NLR	CNL	Chitin-primed	Primed state	A3, A5, RI	Yes
*AT1G63350*	N/A	NLR	CNL	Chitin-primed	Primed state	RI	No
*AT4G16860*	*CHS2, RPP4*	NLR	TNL	Chitin-primed	Primed state	A3, RI, SE	Yes
*AT5G20480*	*EFR*	PRR	RLK	*Pst*-challenged	Secondary challenge	A3, A5, RI, SE	Yes
*AT1G61380*	*LORE, SD1-29*	PRR	RLK	*Pst*-challenged	Secondary challenge	RI	No
*AT1G59218*	*RPP13*	NLR	CNL	*Pst*-challenged and chitin-primed	Secondary challenge and primed state	A3, RI	Yes
*AT1G58848*	N/A	NLR	CNL	*Pst*-challenged and chitin-primed	Secondary challenge and primed state	A3, RI	Yes
*AT3G04210*	*TN13*	NLR	TNL	*Pst*-challenged and chitin-primed	Secondary challenge and primed state	RI	Yes
*AT3G44400*	N/A	NLR	TNL	RS31OX line	Splicing regulator	A3, A5, RI, SE	Yes
*AT1G51800*	*IOS1*	PRR	RLK	*prmt5* mutants	Splicing regulator	RI	Yes
*AT3G44670*	*DM2H*	NLR	TNL	*prmt5* and *ilp1* mutants	Splicing regulator	A3, RI	Yes
*AT1G56540*	*WRR4B*	NLR	TNL	*prmt5* mutants	Splicing regulator	RI	Yes
*AT4G16950*	*RPP5, SIKIC2*	NLR	TNL	*prmt5* mutants	Splicing regulator	RI	Yes
*AT5G46510*	*VICTL*	NLR	TNL	*picln* mutants	Splicing regulator	A3, A5, RI	Yes
*AT1G58807*	N/A	NLR	CNL	*ilp1* mutants	Splicing regulator	RI	Yes
*AT1G58848*	N/A	NLR	CNL	*ilp1* mutants	Splicing regulator	A3, RI	Yes
*AT3G44480*	*RPP1, COG1*	NLR	TNL	*ilp1* mutants	Splicing regulator	A3, A5, RI	Yes
*AT1G61180*	*UNI*	NLR	CNL	*ilp1* mutants	Splicing regulator	RI	No
*AT4G19530*	N/A	NLR	TNL	*ilp1* mutants	Splicing regulator	A5, RI	No
*AT4G16990*	*RLM3*	NLR	TNL	*ilp1* mutants	Splicing regulator	A3, A5, RI, SE	Yes
*AT5G60300*	*DORN1, P2K1*	PRR	RLK	*ddm1* mutants	Chromatin remodeler	RI	No
*AT1G51890*	N/A	PRR	RLK	*ddm1* mutants	Chromatin remodeler	A3, RI	Yes
*AT1G59218*	*RPP13*	NLR	CNL	*ddm1* mutants	Chromatin remodeler	A3, RI	Yes
*AT1G63750*	N/A	NLR	TNL	*ddm1* mutants	Chromatin remodeler	RI	Yes
*AT5G40910*	N/A	NLR	TNL	*ddm1* mutants	Chromatin remodeler	A3, A5, RI, SE	Yes
*AT5G45060*	*RPS4B*	NLR	TNL	*ddm1* mutants	Chromatin remodeler	RI	Yes
*AT4G33300*	*ADR1-L1*	NLR	RNL (helper)	*ddm1* mutants	Chromatin remodeler	RI	Yes
*AT4G16860*	*CHS2, RPP4*	NLR	TNL	*prmt5*, *ilp1* and *ddm1* mutants	Splicing regulator and chromatin remodeler	A3, RI, SE	Yes
*AT1G58602*	*RPP7*	NLR	CNL	*ilp1* and *ddm1* mutants	Splicing regulator and chromatin remodeler	A3, A5, RI	Yes
*AT5G46520*	*VICTR*	NLR	TNL	*ilp1* and *ddm1* mutants	Splicing regulator and chromatin remodeler	A3, RI	Yes

a PRR (Pattern recognition receptor) and NLR (Nucleotide-binding leucine-rich repeat receptor) indicate immune receptor classes.

b TNL (Toll/Interleukin-1), CNL (coiled-coil) and RNL/helper (RPW8-like) indicate NLR subclasses; RLK (receptor-like kinase) indicates a PRR subclass.

c Chitin-primed: indicates unchallenged systemic leaves of root-chitin-primed plants; *Pst*-challenged: indicates pathogen-challenged leaves of root-chitin-primed plants; *prmt*5, *picln*, *rs*31, *ilp*1, *ddm1* indicate RNAseq data from such mutant lines (Makechemu et al., 2025; Mateos et al., 2023; Koster et al., 2025; Wang et al., 2019; Ning et al., 2020).

d Indicates the experimental context in which each gene displayed DAS, including priming phases (primed state or secondary challenge) and genetic perturbations affecting splicing regulators or chromatin remodelers.

N/A: Not available.

e A3 and A5 stand for alternative 3’ and alternative 5’ splice site, RI stand for Intron retention and SE stand for Exon Skipping. No isoform with mutually exclusive exon event were detected for NLRs nor PRRs.

f Indicates if at least one of the isoforms of gene are targets of Nonsense-mediated mRNA decay (NMD) predicted with SUPPA.

These observations suggest that priming maintenance and secondary challenge phases display distinct AS profiles. AS changes in *PRRs* and *NLRs* are not global for the entire families but appear locus-specific for certain immune receptors. However, determining how widespread this mechanism truly is will require gene-by-gene validation of isoform abundance and/or deeper sequencing, including long-read RNA sequencing across multiple systemic resistance and priming programs, to resolve full-length isoforms and detect low-abundance variants.

### 2.5 Splicing regulators as a memory layer

Splicing regulators, including spliceosome components and SR proteins, shape AS patterns and influence immune outputs. Several of these factors modulate the splicing of immune receptors or downstream defense genes, and their disruption frequently alters immune signaling or responsiveness. Mutants affecting spliceosome dynamics, such as *prmt5–picln* (Sanchez et al., 2010; Xu et al., 2011; Mateos et al., 2023), *ntr1* and *ilp1* (Wang et al., 2019), or SR proteins like SR34a, RS31, and SR45 (Zhang et al., 2017b; Laloum et al., 2018; Bui et al., 2025; Koster et al., 2025), among others, alter the expression and/or alternative splicing of specific stress/defense-related signaling components (Palusa et al., 2007; Duque, 2011). Pathogens also hijack elements of the splicing machinery such as WtsE targeting PP2A B′ (Jin et al., 2016), or RipP2 targeting SR34a (Li and Kou, 2025), highlighting the relevance of splicing regulation during infection.

In this context, splicing factors may contribute to priming both by modulating receptor AS and/or by being transcriptionally reprogrammed during priming. RNA-seq datasets support this view: RS31, SR34a, and SR45 influence the splicing or transcript levels of multiple defense genes, including several *PRRs* and *NLRs* (Zhang et al., 2017b). In addition, chitin-triggered priming shows induction of several *SR* genes and down-regulation of *PRMT5*, during primed-state maintenance and secondary challenge (**Supplementary Figure 2**), suggesting that splicing is dynamically altered across the priming process. Thus, to evaluate the extent of potential AS-mediated effects on immune receptors, we intersected our *PRR/NLR* sets with genes showing significant DAS in response to mutations/overexpression in splicing regulators RS31, PRMT5, PICLN and ILP1 (Splicing_DAS, **Figure 1D**). The analysis revealed that most DAS events occur in genes outside the *PRR/NLR* sets, indicating that these regulators primarily reshape the broader transcriptome. Nevertheless, we identified a subset of immune receptors whose splicing was sensitive to these mutants, including 14 NLRs and 1 PRR overlapping with the Splicing_DAS set (**Table 1**). This is consistent with the idea that splicing factors may contribute to a memory layer in systemic resistance and priming. By shaping spliceosome activity and influencing how AS patterns are maintained or reactivated during secondary exposure, splicing regulators could modify isoforms adjustment in a subset of immune receptors (and other defense genes), thereby fine-tuning the responsiveness of primed plants. Future analyses of receptor isoform abundance in splicing-factor mutants and under priming conditions will be required to determine the functional relevance of this potential regulatory mechanism.

### 2.6 Chromatin remodeling and potential effect on PRR/NLR AS

Spliceosome dynamics and RNA polymerase II elongation are tightly coupled to chromatin state (Godoy Herz and Kornblihtt, 2019; Kufel et al., 2022). In addition, *TFIIS* mutants alter the expression of *PRR/NLR* genes (Antosz et al., 2020); **Supplementary Table 6**). Next, we wondered whether chromatin remodelers associated with priming might also influence AS patterns in immune receptors. Remodelers such as MOM1 and DDM1 modulate DNA methylation, nucleosome occupancy, and transcriptional accessibility at immune loci, including *PRR/NLR* clusters (Alvarez et al., 2010; Iwasaki and Paszkowski, 2014; Bourguet et al., 2018; Cambiagno et al., 2018; Furci et al., 2019). Thus, we re-analyzed transcriptomes from the chromatin mutants *mom1* and *ddm1* (**Supplementary Table 5**) and questioned specifically about our *PRR/NLR* sets. This analysis revealed that the epigenetic state correlates with changes in the splicing of immune receptors (Chromatin_DAS, **Figure 1D**). Interestingly, *ddm1* plants showed altered DAS in one *PRR* and in several well-known *NLR* genes, including *ADR1-L1*, a key helper-RNL whose isoform shifts may affect priming (**Table 1**) (Dong et al., 2016; Wu et al., 2021). Conversely, although MOM1 strongly influences priming and regulates *PRR/NLR* chromatin states (Cambiagno et al., 2018; Miranda de la Torre et al., 2023), *mom1* mutant did not affect the AS of any of the *PRRs* or *NLRs* analyzed. This divergence between DDM1 and MOM1 suggests that chromatin remodelers differ in their ability to control co-transcriptional splicing, consistent with the distinct chromatin-related functions of these regulators, particularly the role of DDM1 in regulating transcription through DNA methylation and chromatin compaction (Vaillant et al., 2006; Zemach et al., 2013). In any case, this supports the idea that AS regulation is not a mere by-product of global chromatin deregulation but instead exhibits locus specificity.

Collectively, these analyses suggest that chromatin state and transcription may influence *PRR/NLR* AS, thereby linking epigenetic memory to post-transcriptional regulation. AS changes associated with DDM1 highlight mechanistic specificity among priming-related remodelers and suggest that only a subset of chromatin pathways contributes to receptor AS isoforms tuning during priming.

### 2.7 Exitron splicing in PRR/NLR regulation and its potential role in priming

Finally, beyond canonical AS events, we considered exitron splicing as an additional source of receptor diversification (Marquez et al., 2015; Staiger and Simpson, 2015; Shamnas et al., 2024), particularly for NLRs, which often require fine-tuned domain modulation for signaling. Exitrons are relevant in plants as intron retention is the most frequent alternative splicing event, but it often leads to non-productive transcripts that could trigger nonsense-mediated mRNA decay or generate putative truncated proteins. Exitrons, formerly referred to as cryptic introns (Marquez et al., 2012), lead to RNA variants with full coding potential effectively diversifying the proteome. In fact, exitron retention or removal can subtly alter architecture, post-translational modification motifs, or protein stability (Marquez et al., 2015). A characterized example is the PRR FLS2, whose exitron isoforms modulate receptor abundance and signaling sensitivity (Cheng et al., 2020). Such adjustments may also be especially relevant in priming, where refined variation of the receptor function can enhance responsiveness during secondary challenge.

**Figure 2:**
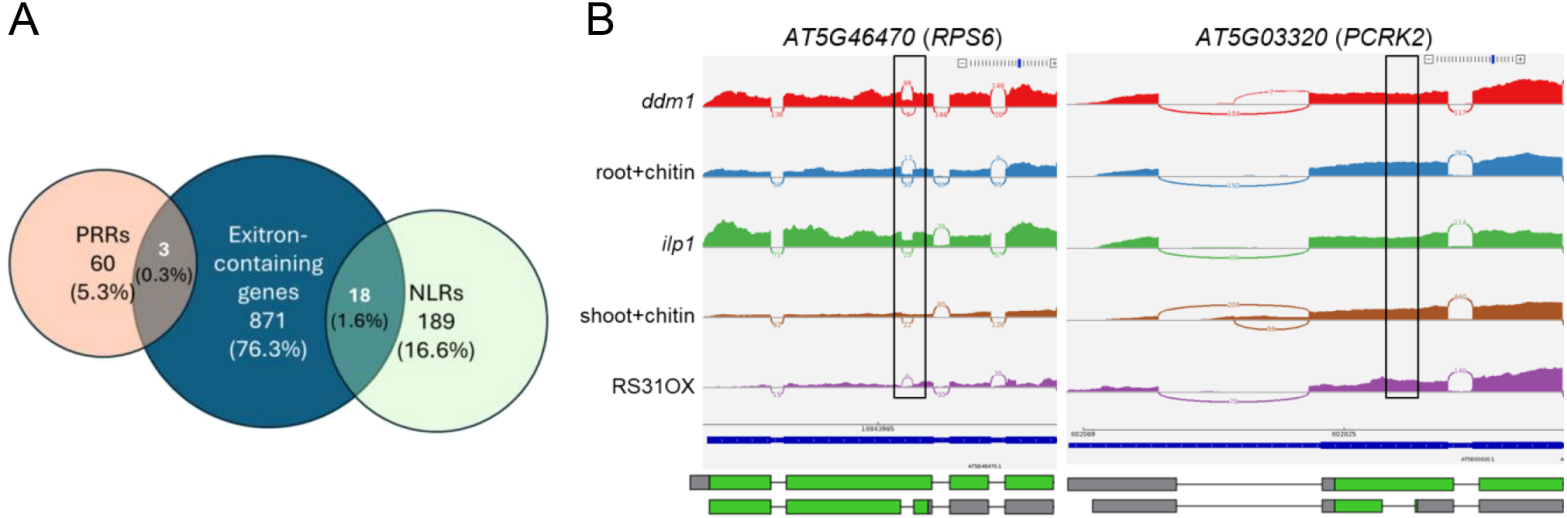
Exitron landscape for immune receptors. **(A)** Venn diagram comparing genes with putative exitrons (Marquez et al., 2015) with NLRs and PRRs listed in **Supplementary Tables 1**-**4**. **(B)** IGV tracks and Sashimi plots for RPS6 (right panel, first four exons) and *PCRK2* (left panel, first three exons). In RPS6, exitron splicing generates a premature termination codon (PTC), whereas in PCRK2 no splice-junction reads were detected across the exitron region. Black rectangles indicate annotated exitron positions (Marquez et al., 2015). Bottom: boxes represent exons, lines represent introns; UTRs are shown in gray and CDSs in green.

To explore this possibility, we searched the Marquez et al. (2015) dataset using our *PRR* and *NLR* sets. We identified multiple NLRs containing predicted exitrons (**Figure 2A**), whereas only three *PRRs*, in addition to *FLS2* (Cheng et al., 2020), show exitron events. This pattern suggests that *NLRs* may be more prone than *PRRs* to generate functional isoforms through exitron splicing, potentially contributing to their structural and regulatory flexibility. Interestingly, one of the predicted exitron-containing *NLRs* is the key helper-*RNL NRG1B* (Castel et al., 2019). Despite this enrichment, our analysis of AtRTD2-based transcriptomes, including primed tissues and mutants of splicing regulators/chromatin remodelers, revealed exitron splicing changes in only a small subset of *NLR* genes, including the receptor *RPS6* (Kim et al., 2009), but not in *PRRs* such as *PCRK2* (**Figure 2B**).

Although clear exitron remodeling in *PRRs* or *NLRs* was not observed, the presence of exitrons in several immune receptors, together with their potential to produce functional isoforms, points to an underexplored regulatory mechanism that may be highly relevant for plant immunity. Future analyses of receptor isoform abundance using additional priming conditions, stimuli, and high-depth transcriptomes may uncover context-specific exitron AS with functional significance.

## 3 Discussion

A defining feature of immune priming is the ability to enhance defensive potential without constitutively activating immunity. AS of *PRRs* and *NLRs* could, in principle, support this poised state because it can adjust receptor properties/levels without requiring major changes in transcriptional regulation. Several lines of evidence illustrate how AS of immune receptors may contribute to priming. First, AS could generate receptor isoforms with altered functional domains, subcellular localization, among others, potentially modifying ligand perception, or modulating signaling strength. Isoform diversity in *PRRs* and *NLRs* has been proposed to shape receptor sensitivity and activation thresholds (Lai and Eulgem, 2018; Hewezi, 2024; Godinho et al., 2025). Second, AS may contribute to control immune receptor abundance. Primed tissues would benefit from accumulating higher levels of receptors or maintaining a “safe” ratio between active, partially active, and compartment-restricted variants. Upon pathogen re-exposure, rapid shifts toward fully active isoforms could support faster or more robust signaling as previously observed (Tateda et al., 2014; Conrath et al., 2015). Third, changes in the activity of splicing regulators during priming may facilitate rapid isoform switching. Stress-responsive regulators such as SR proteins may alter localization, abundance, or post-translational state, affecting spliceosome activity during secondary infection (Jin, 2022). Fourth, intron detention in relevant immune effectors, enhanced by priming, could serve as a sort of molecular memory allowing cells to rapidly generate the necessary coding isoforms upon pathogen attack (Petrillo, 2023).

Within this framework, our *in silico* analysis of AtRTD2 annotations shows that *NLRs* exhibit higher isoform diversity than the average gene (**Figures 1A, B**), suggesting that this gene family might possess an intrinsically elevated capacity for isoform diversification. We detected AS events in both *PRRs* and *NLRs* during primed state and secondary challenge (**Figure 1C**), implying that splicing can potentially modulate immune receptors. These changes are not widespread, affecting ~2.9% of all *PRRs*/*NLRs* or ~4.6% of the expressed receptors suitable for analysis (**Figure 1C**, **Supplementary Table 5**), suggesting that AS regulation under priming is restricted to a subset of immune receptors rather than globally reprogrammed. We also found that AS-regulating components, such as SR proteins, appear to affect the splicing of a limited subset of *PRRs* and *NLRs* (**Figure 1D**), consistent with targeted rather than global effects on receptors RNA processing. Correspondingly, we observed expression changes in several splicing regulators, especially during memory maintenance (**Supplementary Figure 2**), supporting the idea that splicing factors contribute to priming. However, the DAS estimate is constrained by the depth and resolution of available datasets, and additional isoform-level regulation may remain undetected. Even in our highest-quality dataset (Makechemu et al., 2025), ~18% of *NLR* genes were filtered out (**Supplementary Figure 1**; **Supplementary Table 5**), likely reflecting the low basal expression of many NLRs and highlighting the need for deeper, high-coverage datasets with replication and experimental validation. In addition, future studies should prioritize the discovery of novel variants, as the low expression levels typically associated with these genes often hinder their comprehensive annotation.

Interestingly, we identified a potential functional distinction between the chromatin regulators DDM1 and MOM1 in the modulation of receptor transcripts. Although both proteins contribute to immune priming and converge in the repression of transposable elements (Mittelsten Scheid et al., 2002; Iwasaki and Paszkowski, 2014; Cambiagno et al., 2018, 2021; Miranda de la Torre et al., 2023), only DDM1 appears to influence the DAS of *PRR* and *NLR* genes. Because MOM1 lacks canonical remodeling activity and has a more limited impact on global chromatin structure than the SWI2/SNF2-like remodeling functions of DDM1 (Habu et al., 2006; Vaillant et al., 2006; Zemach et al., 2013; Lyons and Zilberman, 2017; Akinmusola et al., 2023; Li et al., 2023), one possibility is that alternative splicing of immune receptors is particularly sensitive to nucleosome dynamics and chromatin accessibility rather than to transcriptional silencing *per se*. In this scenario, DDM1 could modulate AS through transcription-splicing coupling, whereby its chromatin-remodeling activity facilitates a regulatory layer that MOM1-mediated silencing cannot access (Ali et al., 2025). This divergence suggests that, although distinct epigenetic pathways may converge at the level of silencing, the fine-tuning of receptor transcript AS isoforms depends more strongly on chromatin remodeling. Altogether, our *in silico* analyses suggest that splicing factors and chromatin remodelers can shape *PRR* and *NLR* AS isoforms. *Diverse priming* cues, whether genetic, microbial, or abiotic, may reconfigure AS in specific receptor subsets through distinct SR proteins or chromatin remodelers, yet ultimately converge to promote more rapid and robust defense activation upon re-exposure.

Among the major AS modes, two could be particularly relevant for immune receptors, especially in relation to protein diversification and immune memory. Exitron splicing remodels protein domain composition while preserving coding potential (Marquez et al., 2015; Staiger and Simpson, 2015). Consistent with this, we identified several immune receptor genes containing predicted exitrons, whose derived isoforms are more likely to retain functionality and could therefore contribute to defense signaling and priming. Although only one *NLR* showed exitron changes across priming conditions or splicing/chromatin mutants, several predicted key *NLRs* and a subset of *PRRs* harbor exitrons and warrant further investigation (**Figure 2**). Intron retention (IR), the most frequent AS type in plants, represents another distinct AS mode of interest. Although many IR isoforms are degraded, an increasing number corresponds to “detained transcripts” that remain in the nucleus and can be rapidly spliced into mature mRNAs when required (Petrillo, 2023). Interestingly, a recent study identified about 1500 IR events in flg22 treated seedlings (Reyes et al., 2025). Such IR isoforms may function as a post-transcriptional reservoir, enabling swift receptor production during priming or secondary challenge. Nevertheless, the functional contribution of specific IR or exitron-derived receptor isoforms during priming remains to be experimentally validated.

Plant alternative splicing is still a complex scenario. For instance, Howard et al. (2013) suggested that *Pseudomonas* infection triggers AS changes in nearly every Arabidopsis gene. However, this observation relied on the detection of single unaligned reads (inconsistent read pairs), which may be susceptible to technical artifacts. Sequencing-related biases—including 3’ end enrichment, PCR duplication, and alignment software inaccuracies—can generate false positives in AS detection. This underscores the critical necessity of establishing a consensus and also rigorous pipelines for alternative splicing analysis to distinguish genuine biological regulation from technical noise. In addition, based on low levels of expression of some defense genes in normal growth conditions, a more comprehensive *de novo* annotation of isoforms is imperative.

In summary, we propose that alternative splicing may function as a regulatory layer acting on both *PRRs* and *NLRs*, potentially contributing to immune readiness through modulation of isoforms balance. Detained transcripts, isoform-specific localization, dynamic shifts in splicing regulators, and chromatin-AS coupling may collectively provide the flexibility and reversibility characteristics of priming. Overall, AS emerges as a plausible, but still largely untested, component of plant immune memory, emphasizing the need for experimental validations and isoform-level resolution in future RNA-seq studies.

## 4 Future directions, research priorities and validation strategies

Understanding how AS contributes to immune priming and systemic resistance will require integrated molecular and computational efforts. Key priorities include:

- Isoform-level validation of AS events. A necessary first step will be the confirmation of context-dependent AS changes using isoform-specific RT-PCR and targeted quantitative assays. This validation is essential to determine whether predicted priming-associated splice variants of *PRRs* and *NLRs* are reproducibly regulated across biological replicates and conditions.
- Enhanced transcriptomic resources. Gene-by-gene validation of splicing isoform abundance and deeper sequencing, particularly long-read RNA sequencing across multiple priming programs, will be necessary to robustly characterize AS in immune receptors. High-depth, time-resolved priming datasets with diverse stimuli and biological replicates are required to map AS dynamics, an especially critical need for NLRs, whose usually low basal expression makes isoform detection and quantification challenging.
- Multi-omics validation. Integrating RNA-seq with ribosome profiling, proteogenomics, and targeted mass spectrometry could help determine whether specific isoforms are translated in primed versus naïve tissues.
- Improved annotations. Updated At-RTD reference transcriptomes and long-read Iso-Seq data will reveal overlooked receptor isoforms, including rare exitrons.
- Functional testing of isoforms. Many *PRRs* and *NLRs* produce multiple splice variants with unknown roles. Isoform-specific complementation, using native, spliced, and non-spliceable constructs, and compartment-targeted or domain-swap assays, might clarify whether distinct isoforms (e.g. from key receptors such as *NRG1*/*ADR1*, *FLS2*, and *SERK*/*BAK1*) fine-tune defense signaling and/or contribute to immune priming.
- Chromatin-AS integration. Because chromatin state shapes co-transcriptional splicing, future work should determine how priming-associated histone marks, DNA methylation, and elongation rates influence AS at immune loci.
- Exitron-focused analyses. Although not broadly remodeled during priming, exitrons in several *NLRs* and helper *RNLs* may provide a restricted but high-impact regulatory layer. Dissecting how exitron retention/splicing alters domain architecture and priming capacity remains a priority.
- Intron retention as a regulatory reservoir. IR transcripts may serve as post-transcriptional processing substrates, enabling rapid receptor production during secondary challenge. Nuclear fractionation, IR-regulator mutants, and NMD perturbations can test this model.
- Evolutionary comparisons. Cross-species analyses of AS in PRRs, NLRs, and splicing regulators will identify conserved versus lineage-specific modes of AS-mediated priming.

## Data Availability

The original contributions presented in the study are included in the article/**Supplementary Material**. Further inquiries can be directed to the corresponding authors.

## Supplementary material

Supplementary Figure 1 Bioinformatic pipeline used in this study.

Supplementary Figure 2 Expression of splicing-related factors across priming-datasets.

Supplementary Table 1 Arabidopsis annotated and predicted NLRs list from NLRscape.

Supplementary Table 2 Annotated isoforms of NLR genes in ATRTD2 reference transcriptome.

Supplementary Table 3 Arabidopsis PRRs curated and extracted from amiGO.

Supplementary Table 4 Annotated isoforms of PRR genes in ATRTD2 reference transcriptome.

Supplementary Table 5 Datasets used in this study.

Supplementary Table 6 Differential expression and differential alternative splicing in datasets used.
