# Supplementary material for: Alternative splicing: an underexplored layer in immune receptor regulation, systemic resistance and priming
Source: Front Plant Sci. 2026 Mar 13;17:1756671. doi: 10.3389/fpls.2026.1756671 (PMC13021637; doi:10.3389/fpls.2026.1756671)
Supplement: Supplementary Figure 1 — Bioinformatic pipeline used in this study. [file Image1.pdf]

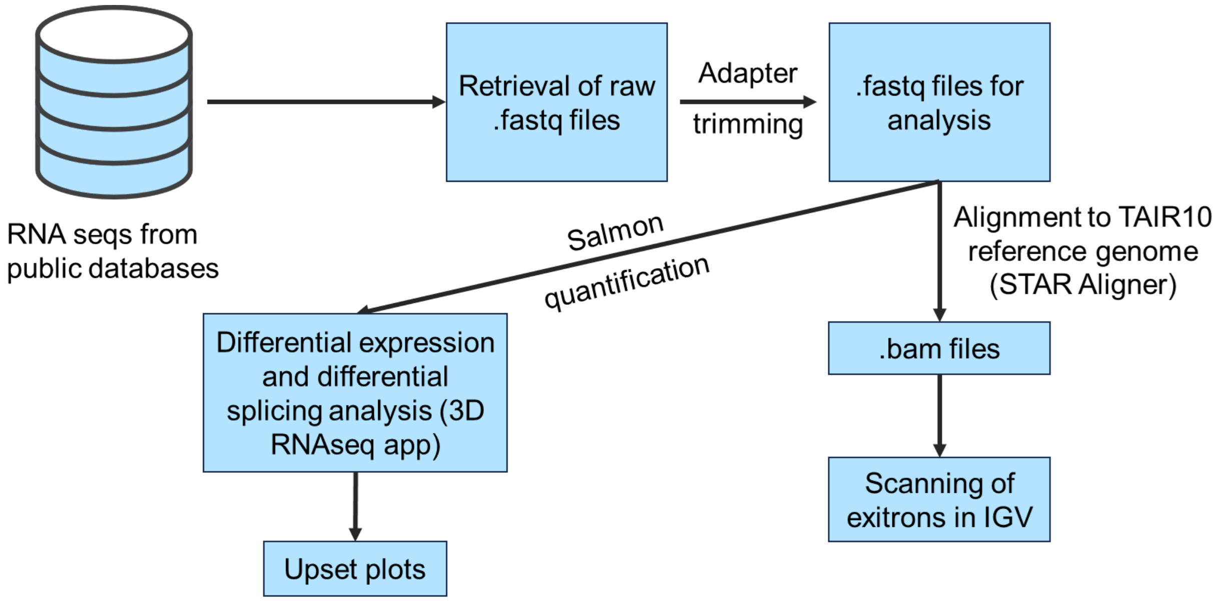

Supplementary figure 1. Bioinformatic pipeline used in this study. Raw RNA data was downloaded from NCBI/SRA. The retrieval of SRA files was done in bash with prefetch - -option-file and converted into fastq with fasterq-dump. Adapter trimming was performed with fastx-toolkit. Once trimmed, reads were quantified with salmon using AtRTD2_QUASI transcriptome reference and the option - -validateMappings. Resulting quant.sf files were used as input from 3D RNAseqApp (https://3drnaseq.hutton.ac.uk/app_direct/3DRNAseq/) for differential gene expression and differential splicing analysis. Output tables from selected data were used for upset plots of Fig. 1C and D. In parallel, trimmed fastq files were aligned to TAIR10 reference genome with STAR Aligner (options -alignSJDBoverhangMin 1 -alignIntronMax 5000 --quantMode GeneCounts). Further details about raw RNA seq data can be found on Supplementary table 5.
