# Supplementary material for: Alternative splicing: an underexplored layer in immune receptor regulation, systemic resistance and priming
Source: Front Plant Sci. 2026 Mar 13;17:1756671. doi: 10.3389/fpls.2026.1756671 (PMC13021637; doi:10.3389/fpls.2026.1756671)
Supplement: Supplementary Figure 2 — Expression of splicing-related factors across priming-datasets. [file Image2.pdf]

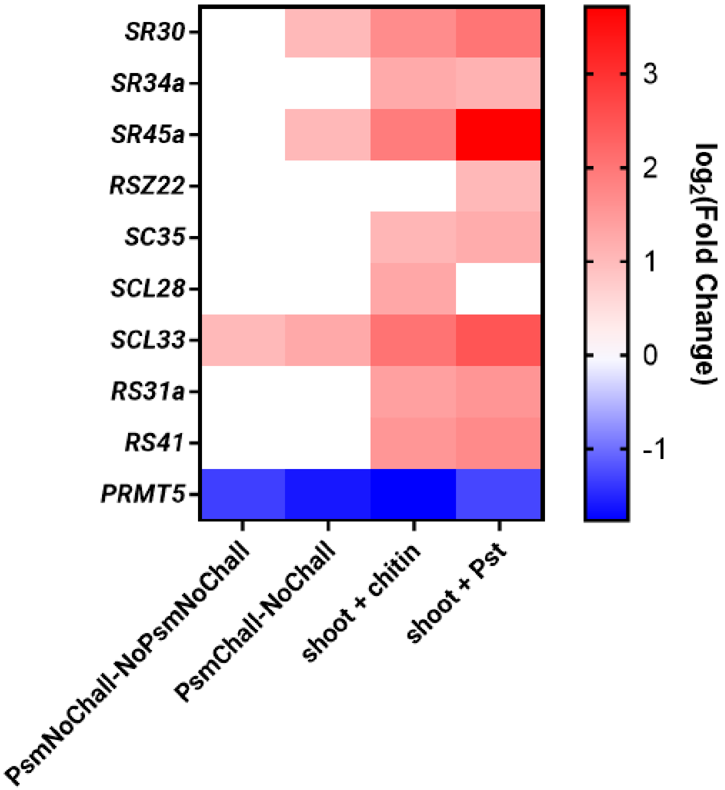

Supplementary figure 2. Splicing-related factors' expression across priming-linked datasets. Log2(Fold Change) from each transcript in each contrast was retrieved from supplementary tables S22, S24, S30 and S32. Blue: upregulated expression; red: Downregulated expression. PsmChall-NoChall: contrast for primed plants vs non-primed plants after challenge. PsmNoChall-NoPsmNoChall: contrast for primed plants vs non primed plants before challenge. Shoot + chitin: Aerial tissue extracted from plants watered with chitin (Induction of ISR). Shoot + *Pst*: Aerial tissue extracted from non-primed plants infected with *Pseudomonas syringae* DC3000.
