# Supplementary material for: Alternative splicing: an underexplored layer in immune receptor regulation, systemic resistance and priming
Source: Front Plant Sci. 2026 Mar 13;17:1756671. doi: 10.3389/fpls.2026.1756671 (PMC13021637; doi:10.3389/fpls.2026.1756671)
Supplement: Supplementary file 5 [file Table2.docx]

Supplementary Table 5. Raw RNA seq datasets used in this study.

| Genotype/Treatment | Bioproject accession number | Sequencing depth^1^ | Type of Sequencing | Expressed NLRs^2^ (% from total) | Expressed PRRs^2^ (% from total) | Article source |
| --- | --- | --- | --- | --- | --- | --- |
| RS31OX/*rs31* | PRJNA367506 | ~170M | paired-end | 152 (73%) | 59 (93%) | Köster, et al. (2025) |
| *sr34a* | PRJNA1044781 | ~85M | paired-end | 110 (53%) | 43 (68%) | Laloum, et al. (2023) |
| SAR: *Psm* + Challenge | PRJEB32929 | ~80M | single-read | 132 (64%) | 59 (93%) | Baum, et al. (2019) |
| ISR: Chitin + *Pst* | PRJNA1253890 | ~68M | paired-end | 171 (82%) | 62 (99%) | Makechemu, et al. (2025) |
| *mom1* | PRJEB28655 | ~36M | single-read | 130 (63%) | 57 (90%) | Bourguet, et al. (2018) |
| *ddm1* | PRJNA566439 | ~30M | paired-end | 136 (66%) | 60 (95%) | Ning, et al. (2020) |
| *picln* and *prmt5* | PRJNA559541 | ~24M | single-read | 134 (65%) | 56 (89%) | Mateos, et al. (2023) |
| PIP | PRJEB43717 | ~22M | single-read | 128 (62%) | 54 (86%) | Yildiz, et al. (2021) |
| NHP | PRJEB43717 | ~22M | single-read | 120 (58%) | 52 (83%) | Yildiz, et al. (2021) |
| SR45OX/*sr45* | PRJNA382852 | ~22M | single-read | 118 (57%) | 45 (71%) | Zhang, et al. (2017) |
| *ntr1* and *ilp1* | PRJNA492822 | ~20M | paired-end | 130 (63%) | 50 (79%) | Wang, et al. (2019) |
| *tfiis* | PRJNA529898 | ~12M | single-read | 131 (64%) | 59 (93%) | Antosz, et al. (2020) |

^1^Average read number across samples was calculated in bash with grep -c ‘@’ *.fastq.

^2^Expressed *NLR*s/*PRRs* were obtained based on genes with CPMs ≥ 2 in at least two samples.

**References**

Antosz, W., Deforges, J., Begcy, K., Bruckmann, A., Poirier, Y., Dresselhaus, T., et al. (2020). Critical Role of Transcript Cleavage in Arabidopsis RNA Polymerase II Transcriptional Elongation. *Plant Cell* 32(5)**,** 1449-1463. doi: 10.1105/tpc.19.00891.

Baum, S., Reimer-Michalski, E.M., Bolger, A., Mantai, A.J., Benes, V., Usadel, B., et al. (2019). Isolation of Open Chromatin Identifies Regulators of Systemic Acquired Resistance. *Plant Physiol* 181(2)**,** 817-833. doi: 10.1104/pp.19.00673.

Bourguet, P., de Bossoreille, S., Lopez-Gonzalez, L., Pouch-Pelissier, M.N., Gomez-Zambrano, A., Devert, A., et al. (2018). A role for MED14 and UVH6 in heterochromatin transcription upon destabilization of silencing. *Life Sci Alliance* 1(6)**,** e201800197. doi: 10.26508/lsa.201800197.

Koster, T., Venhuizen, P., Lewinski, M., Petrillo, E., Marquez, Y., Fuchs, A., et al. (2025). At-RS31 orchestrates hierarchical cross-regulation of splicing factors and integrates alternative splicing with TOR-ABA pathways. *New Phytol* 247(2)**,** 738-759. doi: 10.1111/nph.70221.

Laloum, T., Martín, G., Lewinski, M., Yanez, R.J.R., Köster, T., Staiger, D., et al. (2023). An Arabidopsis SR protein relieving ABA inhibition of seedling establishment represses ABA-responsive alternative splicing. *bioRxiv preprint*. doi: 10.1101/2023.12.19.572415.

Makechemu, M., Goto, Y., Schmid, M.W., Zbinden, H., Widrig, V., Kaufmann, M., et al. (2025). Chitin Soil Amendment Triggers Systemic Plant Disease Resistance Through Enhanced Pattern-Triggered Immunity. *Plant Biotechnol J* 23(11)**,** 5032-5044. doi: 10.1111/pbi.70282.

Mateos, J.L., Sanchez, S.E., Legris, M., Esteve-Bruna, D., Torchio, J.C., Petrillo, E., et al. (2023). PICLN modulates alternative splicing and light/temperature responses in plants. *Plant Physiol* 191(2)**,** 1036-1051. doi: 10.1093/plphys/kiac527.

Ning, Y.Q., Liu, N., Lan, K.K., Su, Y.N., Li, L., Chen, S., et al. (2020). DREAM complex suppresses DNA methylation maintenance genes and precludes DNA hypermethylation. *Nat Plants* 6(8)**,** 942-956. doi: 10.1038/s41477-020-0710-7.

Wang, J., Chen, S., Jiang, N., Li, N., Wang, X., Li, Z., et al. (2019). Spliceosome disassembly factors ILP1 and NTR1 promote miRNA biogenesis in Arabidopsis thaliana. *Nucleic Acids Res* 47(15)**,** 7886-7900. doi: 10.1093/nar/gkz526.

Yildiz, I., Mantz, M., Hartmann, M., Zeier, T., Kessel, J., Thurow, C., et al. (2021). The mobile SAR signal N-hydroxypipecolic acid induces NPR1-dependent transcriptional reprogramming and immune priming. *Plant Physiol* 186(3)**,** 1679-1705. doi: 10.1093/plphys/kiab166.

Zhang, X.N., Shi, Y., Powers, J.J., Gowda, N.B., Zhang, C., Ibrahim, H.M.M., et al. (2017). Transcriptome analyses reveal SR45 to be a neutral splicing regulator and a suppressor of innate immunity in Arabidopsis thaliana. *BMC Genomics* 18(1)**,** 772. doi: 10.1186/s12864-017-4183-7.
